# High-Intensity Accelerations and Decelerations During Intercounty Camogie Match Play

**DOI:** 10.1177/19417381241276016

**Published:** 2024-09-04

**Authors:** John D. Duggan, Paul J. Byrne, Shane Malone, Stephen-Mark Cooper, Jeremy Moody

**Affiliations:** † Department of Sport, Exercise and Nutrition, School of Science and Computing, Atlantic Technological University, (ATU), Galway Campus, Galway, Ireland; ‡ School of Sport and Health Sciences (Sport), Cardiff Metropolitan University, Cyncoed Campus, Cardiff, Wales; § Department of Health and Sport Sciences, South East Technology University, Carlow Campus, Carlow, Ireland; ‖ School of Physical Education and Sports, Nisantasi University, Istanbul, Turkey; ¶ Gaelic Sports Research Centre, Technological University Dublin-Tallaght Campus, Tallaght, Dublin, Ireland

**Keywords:** accelerations, decelerations, female Gaelic games, injury reduction, player monitoring

## Abstract

**Background:**

This study aimed to compare acceleration and deceleration demands of intercounty Camogie players, and differences across playing positions and halves of play.

**Hypothesis:**

The middle 3 positions will have greatest accelerations and decelerations variables across match play and halves of play.

**Study Design:**

Nonrandomized, repeated measures design.

**Level of Evidence:**

Level 4.

**Methods:**

Global positioning systems (GPS) (10 Hz) collected data from 28 participants during 18 competitive matches across 2 seasons; 206 individual player datasets were analyzed.

**Results:**

Half-backs (*P* < 0.05; effect size [ES], -1.75) and midfielders (*P* < 0.05; ES, -1.68) covered significantly greater total number of accelerations than full-forwards. In acceleration zone 4, midfielders (*P* < 0.05; ES, = -1.67) and half forwards covered a significantly greater number than full-forwards (*P* < 0.01; ES, = -1.41). Midfielders accumulated a significantly greater distance in acceleration zone 4 than full-backs (*P* < 0.05; ES, = -0.57). Significant decrements were observed between halves in total number of accelerations (*P <* 0.01; ES, = 0.49), accelerations in zones 1 to 4 (*P <* 0.01; ES, 0.16-0.43), total distance of accelerations, and acceleration distance in zones 2 to 4 (*P <* 0.05; ES, 0.25; *P <* 0.01; ES, 0.45; *P <* 0.01; ES, 0.38). There were significant decrements in the total number of decelerations (*P <* 0.01; ES, 0.43), number of decelerations in zones 2 (*P <* 0.05; ES, 0.25), 3 (*P <* 0.01; ES, 0.45), and 4 (*P <* 0.01; ES, 0.38), and total deceleration distance (*P <* 0.01; ES, 0.16).

**Conclusion:**

Half-backs and midfielders covered significantly greater total number of accelerations than full-forwards. Significant decrements in several acceleration and deceleration variables were observed between halves.

**Clinical Relevance:**

Players competing in intercounty Camogie should receive progressive exposure to acceleration and deceleration-based movement demands to prepare players for intercounty Camogie match play.

Camogie is an intermittent, invasion-based female field sport in which a team includes a goalkeeper, 2 lines of 3 defensive players (full-backs and half-backs), 2 midfielders, and 2 lines of attacking players (half-forwards and full-forwards).^12^ Match play at elite intercounty level comprises 2 30-minute halves played on a rectangular pitch (145 meter length and 90 meter width).^11^ In Camogie, an ash stick (hurley) is used to strike the slíotar over variable distances. The skills of Camogie include high catching and fielding, aerial duels, striking the sliotar, running with the slíotar on the hurley, blocking opponents with the hurley, and intercepting the slíotar with the hurley or by hand.^12^ During the competitive season, elite intercounty Camogie players compete in 2 major competitions, the National League, and the All-Ireland Championship. During the peak competitive phases, female camogie players may partake in games on a weekly or fortnightly basis depending upon progress through both major competitions.^11^

Global positioning system (GPS) technology has become ubiquitous amongst intercounty Camogie teams.^13^ GPS can be used to objectively quantify the demands of specific competitions and monitor physiological and biomechanical fluctuations in both training and match play.^13,41^ Despite the increased utilization of GPS in female Gaelic games, there remains a dearth of information regarding high-intensity actions in match-play activities. Due to the high velocity, multidirectional, contact nature of Gaelic team sports, female players are inherently at risk of injury.^11^ Athletic movements such as cutting and landing require quick execution and increased forces that need to be produced in a short timeframe, which could lead to ligament rupture.^46^ Previous research conducted in intercounty Camogie reported that lower limb injuries were the most frequent (71.4%), with 23.8% of injuries occurring to the thigh, knee (19%), and ankle (9.5%), with 19% of injuries occurring as a result of sprinting and 14.3% from a change of direction.^4,34^

Due to the intermittent nature of team sports, athletes are required to change speeds frequently.^44^ Accelerations and decelerations are locomotive actions that require rapid changes in direction in addition to velocity and these movements are energetically costly and taxing on the neuromuscular system.^18,26,31^ These actions are both important components of match play in female team-based sports, contributing to increased mechanical stress levels, and overall biomechanical load, which can impact performance potential significantly.^19,39^ As the evolution of female teams sports continues from both technical and tactical perspectives, there will likely be an increase in these high-intensity actions.^21^ It is therefore important to understand the demands of these actions to appreciate how best to prepare female team sport athletes to ensure their health and wellbeing.^16^ It has also been suggested that accelerations have an increased metabolic cost, whereas decelerations have a high mechanical output such as larger ground-reaction forces (GRFs) that increase biomechanical loading.^18,31^ From a mechanical perspective, early acceleration is associated with the ability to produce GRFs in short timeframes and directing the GRFs in a horizontal direction.^33^

Decelerations have a specific ground-reaction profile that is characterized by high impact peak forces and loading rates.^18^ During the penultimate step of maximal decelerations, GRFs have been reported to be 2.7 times greater than the equivalent actions during acceleration.^18^ These actions occur over short timeframes, which lead to increased forces that need to be attenuated during decelerative actions.^43^ Furthermore, match-play acceleration and deceleration performance profiles have been associated with increased creatine kinase activity, reduced neuromuscular performance, and increased perceived self-reported muscle soreness in male team sport athletes.^8^ The qualification of accelerations and decelerations in match play may enable practitioners to monitor and design training interventions to optimize onfield performance and reduce the likelihood of injury in female team sports athletes.^30^ From a technical and tactical perspective, the ability to accelerate and decelerate is a desirable attribute in match play that facilitates movement patterns to transition from defense to attack or vice versa.^37^ It is vital that players can attenuate and distribute high-impact forces through the muscular tendon structure of the lower limb during deceleration-based movements.^19,31^

There has been limited research into acceleration and deceleration activities in female sports.^16,29,30^ In intercounty Ladies Gaelic Football (LGF), players completed an average of 42 ± 6 accelerations of ≥3 m s^−2^, and 53 ± 9 decelerations of ≤3 m s^-2^ during competitive match play.^29^ Furthermore, there were significant reductions in number of accelerations (n) and decelerations (m) (effect size [ES], 0.59-1.20) across halves of play.^29^ In elite female soccer, players performed a total of 423 ± 126 accelerations and 430 ± 125 decelerations during match play, which varied across position. ^30^ The mean and maximum distances per effort ranged between 1 and 4 meters and 2 and 8 meters, respectively. In international female soccer, players covered significantly greater mean acceleration distances and durations at >4 m s^−2^ (39.4 ± 3.5 vs 31.6 ± 6.8) compared with domestic-based players.^16^

There is also limited research into intercounty male hurling, which has demonstrated that players covered 126 ± 25 accelerations (>2 m s^−2^) and 120 ± 26 decelerations (<2 m s^−2^) during match play.^47^ Furthermore, there were significant differences in both accelerations (66 ± 13 vs 61 ± 18 n) and decelerations (63 ± 14 vs 58 ± 18 n) between halves.^47^ Acceleration and deceleration positional demands also differed as half-backs performed a greater number of accelerations to midfielders and full-forwards (142 ± 24 n) and half-backs also performed a greater number of decelerations than all other positions (142 ± 24 n).^47^ Although there is currently no comparable data available in Camogie, knowledge of such specific information can confirm a requirement for increased development of enhanced braking strength capabilities, which may, in turn, enable female GAA players to tolerate high-intensity match play more effectively, which may provide greater resilience to sports-specific injuries.^10,11^ The quantification of both accelerations and decelerations actions in match play can aid practitioners in the design of appropriate training interventions to optimally prepare the athletes to cope with these repetitive increased mechanical and physiological stresses in training and match play. The aim of the current investigation was to establish the demands of acceleration and deceleration of intercounty Camogie match play. It was hypothesized that the middle 3 positions (half-backs, midfielders, and half-forwards) would cover the greatest acceleration and deceleration frequencies and distances across match play and halves of play. It was also hypothesized that there would be a decrement in high-intensity variables across halves of play.

## Methods

### Participants

The n = 28 female outfield intercounty level Camogie players (mean [± SD]: age, 27 ± 4 years; height, 169 ± 5 cm; and body mass, 67 ± 8 kg) participated voluntarily in the current study. Before data collection, participants attended a briefing session where they were informed of the purpose, benefits, and procedures of the study, and written informed consent and medical declarations were obtained from all participants in line with the procedures set out by the University’s research ethics committee in accordance with the Declaration of Helsinki (reference PGR-3036). As part of this observational study, players completed 3 field-based sessions (technical and tactical), and 2 gym-based sessions per week.

### Procedures

This observational study was devised to investigate the acceleration and deceleration demands of elite level intercounty Camogie players using GPS technology across playing positional lines, and halves of match play. Intercounty Camogie players (n = 28), who were part of a senior training panel, were observed over a total of n = 18 competitive games during both the 2019/2020 and 2020/2021 seasons. Only competitive league and championship game match play was considered for analysis. Players were categorized based on 5 positional lines of play (full-backs, half-backs, midfielders, half-forwards, and full-forwards). The number of datasets per position were assessed and included: full-backs, n = 53; half-backs, n = 54; midfielders, n = 37; half-forwards, n = 29, and full-forwards, n = 33. Match-play data were only included if players completed a full game (~60 minutes). All competitive matches took place between 12:00 and 21:00, and the environmental temperature during match play ranged between 4.0°C and 25°C. GPS was used to determine the acceleration and deceleration actions during competitive match play. All players were requested to refrain from strenuous physical activity for 24 hours before competitive match play, and to abide by the pre-event nutritional strategies typical of elite female Gaelic sports teams.^29^

During the initial familiarization session, the players’ stature and body mass was measured using a portable stadiometer (Seca 217, Seca Ltd) and weighing scales (Tanita BC545N, Tanita Ltd), respectively. Both measurements were made without footwear and wearing minimal gym clothing. To determine the acceleration and deceleration demands of match play, participants were required to wear an individual GPS unit (VX110 Log; Visuallex Sport International Ltd) during every competitive game. The GPS unit (mass, 50 g; dimensions, 74 × 47 × 317 mm; sampling, 10 Hz) was enclosed in a bespoke smartvest lying in the upper thoracic region between the scapulae. Each player wore the same GPS unit throughout the 18 matches in both campaigns to ensure interunit reliability.^40^ To obtain an optimal satellite signal, the units were switched on 30 minutes before the start of the warm-up.^15^ It has been demonstrated previously that when sampling at 10 Hz, GPS units are valid for providing metrics such as running distances and velocities in both linear and team-sports-specific circuits.^36^ The validity and reliability of the GPS units have been described previously in the literature.^5,16^

Data collected and derived by the GPS units included 4 acceleration and deceleration zones. Predetermined acceleration and deceleration thresholds were utilized where zone 1 was set between 0.7 and 1.4 m s^-2^, zone 2 between 1.41 and 2.2 m s^-2^, zone 3 between 2.21 and 2.9 m s^-2^, zone 4 >3 m s^-2^.^24^ Other variables included in the observational study were (1) acceleration zone total (n + m), (2) acceleration zones 1 to 4 (n + m), (3) deceleration zone total (n + m), and (4) deceleration zones 1 to 4 (n + m). The minimum instant acceleration to start an event was 0.5 m s^-2^, the minimum speed increase of an acceleration event was 3 km hour^-1^, the minimum distance of a valid acceleration event was 2 meters, and the minimum length of a valid acceleration event was 0.5 m s^-2^. The minimum instant deceleration to start an event was 0.3 m s^-2^, the minimum speed increase of a deceleration event was 2 km hour^-1^, the minimum distance of a valid deceleration event was 2 meters, and the minimum length of a valid deceleration event was 0.3 m s^-2^. Previously, a minimum effort duration (MED) of 0.2 seconds has been utilized during team-based sports.^3,16^ The accelerations and decelerations velocities cited herewith correspond with those summarized in previous research conducted in samples of players from female team sports.^15,29^ The reliability of 10 Hz GPS technology to measure accelerations (coefficient of variation [CV], 1.9-4.3%) and deceleration (CV 6%), displayed good-to-moderate interunit reliability.^7,36^ Once each match was completed, the players’ GPS data were downloaded from each unit retrospectively using the manufacturer’s software (VX Sport Version 5.4.3.63) Each data file was “trimmed” so that only data recorded during each half of each match ie, when the players were actually on the field of play, was included for further analysis. Downloaded data were then exported into a Microsoft Excel file for further analysis.

### Statistical Analyses

Variables are summarized and presented as means and standard deviations together with their 95% CIs. The hypothesis that both acceleration and deceleration variables (dependent variables [DV]) would decrease between halves of play (independent variable 1 [IV]), and that they would be position specific (independent variable 2 [IV2]) was tested using a mixed-design analysis of variance (ANOVA). The level of statistical significance was set at *P* ≤ 0.05 throughout all stages of the analyses. When significant main effects were identified, Bonferroni corrected *t* tests were applied post hoc to establish differences between the 5 levels of playing positions (full-backs, half-backs, midfielders, half-forwards, and full-forwards), and between the 2 halves of play (first half and second half). ESs for the ANOVA outcomes were estimated using partial eta squared (*η*^2^); interpreted as follows: trivial, ≤0.20; small, 0.21 to 0.60; medium, 0.61 to 1.20; large, 1.21 to 2.00, and very large, 2.01 to 4.00.^22^ The ES for each significant difference (*t* test) outcome between sample means was estimated using Cohen’s *d*. For IV1, *d* = _Δ_/*s*_Δ_; and for IV2, *d* = _Δ_/*s*_P_, where _Δ_ = difference between sample means, *s*_Δ_ = SD for _Δ_, and *s*_P_ = pooled SD (*s*_P_ = √((*s*_1_^2^(*n*_1_ - 1) + *s*_2_^2^(*n*_2_ - 1))/(*n*_1_ + *n*_2_ - 2)), where *s*_1_ = SD for sample 1, *s*_2_ = SD for sample 2, *n*_1_ = size of sample 1, and *n*_2_ = size of sample 2). All statistical analyses were performed using SPSS for Windows (Version 28; SPSS, Inc).

## Results

Tables 1 to 4 present summary statistics for the acceleration and deceleration data for total match play, during both first and second halves, and the mean differences between the halves (first half minus second half). The results demonstrated that, during match play, intercounty Camogie players performed a (mean ± SD) total number of 124 ± 29 accelerations and 83 ± 23 decelerations, with the highest number of accelerations occurring in zone 3 (48 ± 13 n) and the highest number of decelerations in zone 3 (26 ± 8 n). Camogie players performed a total distance in accelerations of 202 ± 28 m and 64 ± 11 m in decelerations, with the highest total distance in zone 3 in accelerations of 62 ± 11 m and 18 ± 4 m in decelerations in both zones 3 and 4.

**Table 1.** Totals and differences in first half minus second half, and ES for accelerations for total match play and first and second halves

Variable (unit)	Total^a^	Total First Half^a^	Total Second Half^a^	Difference, First Half - Second Half (95% CI)	ES
Acceleration total (n)	124 ± 29	66 ± 17	58 ± 19**	-9 (-19 to 1)	-0.49 (small)
Acceleration zone 1 (n)	4 ± 5	2.2 ± 3	2 ± 3*	0 (-2 to 1)	-0.16 (trivial)
Acceleration zone 2 (n)	32 ± 13	17 ± 8	15 ± 7**	-2 (-7 to 2)	-0.31 (small)
Acceleration zone 3 (n)	48 ± 13	26 ± 8	22 ± 9**	-3 (-8 to 2)	-0.39 (small)
Acceleration zone 4 (n)	40 ± 11	21 ± 7	18 ± 6**	-3 (-7 to 1)	-0.43 (small)
Acceleration total (m)	202 ± 28	104 ± 20	99 ± 22**	-5 (-17 to 7)	-0.23 (small)
Acceleration zone 1 (m)	27 ± 14	14 ± 10	13 ± 11*	-2 (-8 to 4)	-0.18 (trivial)
Acceleration zone 2 (m)	56 ± 11	28 ± 8	28 ± 9*	-1 (-5 to 4)	-0.08 (trivial)
Acceleration zone 3 (m)	62 ± 11	31 ± 7	31 ± 8	0 (-5 to 4)	-0.06 (trivial)
Acceleration zone 4 (m)	58 ± 10	30 ± 8	28 ± 7*	-2 (-6 to 3)	-0.23 (small)

ES, effect size.

a Data are presented as mean ± SD.

* Difference significant at *P* ≤ 0.05.

** Difference significant at *P* ≤ 0.01.

**Table 2.** Totals and differences in first half minus second half, and ES for decelerations for total match play, first and second halves

Variable (unit)	Total^a^	Total First Half^a^	Total Second Half^a^	Difference, First Half - Second Half (95% CI)	ES
Deceleration total (n)	83 ± 23	44 ± 14	39 ± 14**	-6 (-14 to 2)	-0.43 (small)
Deceleration zone 1 (n)	10 ± 7	6 ± 4	5 ± 4	-1 (-3 to 2)	-0.16 (trivial)
Deceleration zone 2 (n)	28 ± 11	15 ± 6	13 ± 6*	-2 (-5 to 2)	-0.25 (small)
Deceleration zone 3 (n)	26 ± 8	14 ± 5	12 ± 5*	-2 (-5 to 1)	-0.45 (small)
Deceleration zone 4 (n)	18 ± 6	10 ± 4	9 ± 4**	-1 (-4 to 1)	-0.38 (small)
Deceleration total (m)	64 ± 11	33 ± 8	32 ± 8**	-1 (-6 to 3)	-0.16 (trivial)
Deceleration zone 1 (m)	12 ± 4	6 ± 3	6 ± 3	0 (-2 to 1)	-0.12 (trivial)
Deceleration zone 2 (m)	17 ± 4	8 ± 3	8 ± 3	0 (-2 to 2)	-0.03 (trivial)
Deceleration zone 3 (m)	18 ± 4	9 ± 3	9 ± 3	0 (-2 to 1)	-0.12 (trivial)
Deceleration zone 4 (m)	18 ± 4	9 ± 3	9 ± 3	0 (-2 to 1)	-0.17 (trivial)

ES, effect size.

a Data are presented as mean ± SD.

* Difference significant at *P* ≤ 0.05.

** Difference significant at *P* ≤ 0.01.

**Table 3.** Totals and differences as first half minus second half, and ES for accelerations as total, first, and second half accelerations per positional line^a^

	Half	FB	HB	M	HF	FF
Acceleration total (n)	Total	115 ± 28	132 ± 20	139 ± 27	134 ± 31	98 ± 20
	First half	61 ± 14	71 ± 10^b^	75 ± 18^b^	75 ± 15^b^	49 ± 14^c,d,f^
	Second half	54 ± 18	61 ± 16	64 ± 20^b^	59 ± 22	49 ± 12^b^
	95% CI (first to second)	-7 ( -17 to 2)	-9 (-17 to -1)	-11 (-22 to 0)	-16 (-27 to 5)	1 (-7 to 8)
	ES	-0.44 (small)	-0.68 (moderate)	-0.56 (small)	-0.85 (moderate)	0.04 (trivial)
Acceleration zone 1 (n)	Total	3 ± 8	6 ± 3	5 ± 3	5 ± 5	3 ± 2
	First half	2 ± 4	3 ± 2	2 ± 1	3 ± 3	2 ± 1
	Second half	2 ± 4	3 ± 2	3 ± 2	2 ± 2	1 ± 1
	95% CI (first to second)	0 ( -3 to 2)	-1 (-2 to 1)	0 (-1 to 1)	-2 (-3 to 0)	0 (-1 to 1)
	ES	-0.06 (trivial)	-0.33 (small)	0.20 (small)	-0.58 (small)	0.05 (trivial)
Acceleration zone 2 (n)	Total	28 ± 12	39 ± 10	34 ± 14	36 ± 12	22 ± 8
	First half	15 ± 7	21 ± 6^b^	17 ± 8^b^	20 ± 7^b^	11 ± 5^c,d,e^
	Second half	13 ± 7	18 ± 7	17 ± 8^b^	16 ± 7	11 ± 5
	95% CI (first to second)	-2 ( -6 to 2)	-4 (-7 to 0)	0 (-5 to 5)	-5 (-9 to 1)	0 (-3 to 3)
	ES	-0.33 (small)	-0.58 (small)	0.00 (trivial)	-0.65 (small)	0.05 (trivial)
Acceleration zone 3 (n)	Total	44 ± 11	50 ± 9	55 ± 12	54 ± 15	37 ± 8
	First half	23 ± 6	26 ± 6	30 ± 8^b^	30 ± 8^b^	18 ± 5^d,e^
	Second half	21 ± 7	24 ± 8	25 ± 9	24 ± 10	19 ± 6
	95% CI (first to second)	-2 ( -6 to 1)	-2 (-6 to 2)	-5 (-10 to 0)	-7 (-12 to -2)	1 (-3 to 4)
	ES	-0.38 (small)	-0.33 (small)	-0.58 (small)	-0.75 (moderate)	0.14 (trivial)
Acceleration zone 4 (n)	Total	40 ± 11	37 ± 9	47 ± 13	39 ± 10	36 ± 7
	First half	21 ± 6	20 ± 6	27 ± 9	21 ± 6	18 ± 6
	Second half	19 ± 6	17 ± 6	20 ± 8	18 ± 7	18 ± 4
	95% CI (first to second)	-2 ( -6 to 2)	-3 (-6 to 1)	-6 (-11 to -1)	-3 (-7 to 1)	-1 (-3 to 2)
	ES	-0.32 (small)	-0.47 (small)	-0.72 (moderate)	-0.46 (small)	-0.12 (trivial)
Acceleration total (m)	Total	190 ± 30	218 ± 27	207 ± 23	214 ± 27	200 ± 26
	First half	92 ± 18	110 ± 19	103 ± 17	114 ± 22^b^	99 ± 16^c,d,f^
	Second half	86 ± 17	108 ± 20	104 ± 23	100 ± 20	101 ± 19
	95% CI (first to second)	-6 (-16 to 4)	-5 (-16 to 6)	1 (-10 to 13)	-14 (-26 to -2)	0 (-10 to 10)
	ES	-0.33	-0.25	0.07	-0.65 (moderate)	0.02 (trivial)
Acceleration zone 1 (m)	Total	23 ± 14	36 ± 12	22 ± 13	33 ± 14	25 ± 16
	First half	10 ± 9	19 ± 9	11 ± 10	19 ± 9	13 ± 10
	Second half	9 ± 9	17 ± 10	11 ± 13	14 ± 12	12 ± 11
	95% CI (first to second)	-1 (-6 to 4)	-2 (-8 to 3)	0 (-7 to 7)	-5 (-11 to 1)	-2 (-8 to 4)
	ES	-0.12 (trivial)	-0.25 (small)	-0.01 (trivial)	-0.44 (small)	-0.15 (trivial)
Acceleration zone 2 (m)	Total	54 ± 12	59 ± 10	58 ± 9	60 ± 11	53 ± 10
	First half	27 ± 7	29 ± 7	28 ± 6	32 ± 10	25 ± 7
	Second half	24 ± 8	30 ± 11	30 ± 8	28 ± 6	28 ± 7
	95% CI (first to second)	-3 (-7 to 2)	1 (-5 to 6)	1 (-3 to 6)	-4 ( -8 to 1)	2 (-2 to 6)
	ES	-0.35 (small)	0.07 (trivial)	0.18 (trivial)	-0.45 (small)	0.23 (small)
Acceleration zone 3 (m)	Total	59 ± 11	63 ± 12	67 ± 9	63 ± 9	59 ± 13
	First half	29 ± 7	32 ± 7	32 ± 7	33 ± 7	29 ± 8
	Second half	27 ± 7	32 ± 7	35 ± 8	30 ± 7	30 ± 7
	95% CI (first to second)	-2 (-6 to 2)	0 (-4 to 4)	2 (-2 to 7)	-3 (-7 to 1)	1 (-3 to 5)
	ES	-0.27 (small)	-0.04 (trivial)	0.33 (small)	-0.43 (small)	0.12 (trivial)
Acceleration zone 4 (m)	Total	54 ± 11	59 ± 9	60 ± 10	58 ± 11	62 ± 10
	First half	27 ± 7	31 ± 7	31 ± 7	30 ± 9	31 ± 7
	Second half	26 ± 6	28 ± 6	29 ± 7	28 ± 7	31 ± 9
	(95% CI) [1st-2nd]	0 (-4 to 3)	-3 (-7 to 1)	-2 (-7 to 2)	-2 (-7 to 2)	-1 (-5 to 4)
	ES	-0.05 (trivial)	-0.44 (small	-0.30 (small	-0.28 (small	-0.09 (trivial)

ES, effect size; FB, full-back; FF, full-forward; HB, half-back; HF, half-forward; M, midfielder.

a Data are presented as mean ± SD, mean differences as first half minus second half (95% CI).

b Significance difference from FF.

c Significance difference from FB.

d Significance difference from M.

e Significance difference from HF.

f Significance difference from HB.

**Table 4.** Totals and differences as first half minus second half, and ES for total decelerations, first and second half decelerations per positional line^a^

	Half	FB	HB	M	HF	FF
Deceleration total (n)	Total	75 ± 25	91 ± 16	92 ± 24	88 ± 21	65 ± 19
	First half	40 ± 14	49 ± 8	50 ± 14	49 ± 12	32 ± 12
	Second half	35 ± 14	42 ± 12	42 ± 17	38 ± 16	33 ± 9
	95% CI (first to second)	-4 (-12 to 3)	-7 (-13 to -1)	-7 (-16 to 2)	-11 (-19 to -3)	0 (-6 to 7)
	ES	-0.32 (small)	-0.66 (moderate)	-0.47 (small)	-0.77 (moderate)	0.05 (trivial)
Deceleration zone 1 (n)	Total	9 ± 8	13 ± 6	10 ± 8	11 ± 6	8 ± 5
	First half	5 ± 4	7 ± 4	5 ± 4	6 ± 4	4 ± 3
	Second half	4 ± 4	6 ± 3	5 ± 4	5 ± 4	4 ± 3
	95% CI (first to second)	-1 (-3 to 2)	-1 (-3 to 1)	0 (-3 to 2)	-1 (-3 to 2)	0 (-2 to 2)
	ES	-0.17 (trivial)	-0.34 (small)	-0.06 (trivial)	0.24 (small)	0.02 (trivial)
Deceleration zone 2 (n)	Total	25 ± 11	33 ± 7	30 ± 11	31 ± 10	19 ± 8
	First half	13 ± 7	18 ± 4	16 ± 6	17 ± 6	9 ± 4
	Second half	12 ± 6	15 ± 6	14 ± 7	14 ± 7	10 ± 4
	95% CI (first to second)	-1 ( -4 to 3)	-2 (-5 to 1)	-2 (-6 to 2)	-3 (-7 to 1)	0 (-2 to 3)
	ES	-0.10 (trivial)	-0.46 (small)	-0.31 (small)	-0.15 (trivial)	0.10 (trivial)
Deceleration zone 3 (n)	Total	25 ± 7	26 ± 6	30 ± 9	27 ± 9	21 ± 6
	First half	13 ± 5	14 ± 4	17 ± 6^b^	16 ± 5	11 ± 4^c^
	Second half	12 ± 5	12 ± 4	13 ± 6	11 ± 6	10 ± 3
	95% CI (first to second)	-2 (-4 to 1)	-1 (-4 to 1)	-3 (-7 to 0)	-5 (-8 to -2)	-1 (-3 to 2)
	ES	-0.36 (small)	-0.35 (small)	-0.53 (small)	-0.46 (small)	-0.19 (trivial)
Deceleration zone 4 (n)	Total	16 ± 5	19 ± 5	22 ± 7	19 ± 5	17 ± 6
	First half	9 ± 3	10 ± 3	12 ± 5	11 ± 4	8 ± 4
	Second half	7 ± 3	8 ± 3	10 ± 5	8 ± 4	9 ± 3
	95% CI (first to second)	-1 (-3 to 1)	-2 (-4 to 0)	-2 (-4 to 1)	-2 (-5 to 0)	1 (-1 to 3)
	ES	-0.43 (small)	-0.58 (small)	-0.37 (small)	-0.88 (moderate)	0.20 (small)
Deceleration total (m)	Total	64 ± 12	73 ± 11	63 ± 10	61 ± 8	64 ± 12
	First half	29 ± 8	37 ± 7	33 ± 9	32 ± 8	31 ± 8
	Second half	27 ± 7	36 ± 8	30 ± 8	29 ± 7	33 ± 7
	95% CI (first to second)	-2 (-7 to 2)	-1 (-5 to 4)	-4 (-8 to 1)	-1 (-5 to 3)	2 (-3 to 6)
	ES	-0.28 (small)	-0.09 (trivial)	-0.41 (small)	-0.65 (moderate)	0.21 (small)
Deceleration zone 1 (m)	Total	12 ± 4	14 ± 3	11 ± 4	11 ± 3	12 ± 4
	First half	5 ± 3	7 ± 2	6 ± 4	5 ± 3	6 ± 3
	Second half	5 ± 3	7 ± 3	5 ± 3	6 ± 3	6 ± 3
	95% CI (first to second)	-1 (-2 to 1)	0 (-2 to 1)	-1 (-3 to 1)	0 (-1 to 2)	0 (-1 to 2)
	ES	-0.22 (small)	-0.14 (trivial)	-0.36 (small)	-0.13 (trivial)	0.07 (trivial)
Deceleration zone 2 (m)	Total	16 ± 5	19 ± 4	17 ± 5	16 ± 4	15 ± 4
	First half	8 ± 3	9 ± 3	9 ± 3	9 ± 3	7 ± 2
	Second half	8 ± 3	10 ± 4	8 ± 3	7 ± 2	8 ± 3
	95% CI (first to second)	0 (-2 to 2)	1 (-1 to 3)	-1 (-3 to 1)	-1 (-2 to 1)	1 (-1 to 2)
	ES	-0.08 (trivial)	0.18 (trivial)	-0.34 (small)	-0.13 (trivial)	0.25 (small)
Deceleration zone 3 (m)	Total	18 ± 3	20 ± 4	18 ± 4	17 ± 4	18 ± 6
	First half	9 ± 2	10 ± 3	10 ± 3	9 ± 3	9 ± 3
	Second half	9 ± 3	10 ± 3	9 ± 3	8 ± 3	9 ± 3
	95% CI (first to second)	0 ( -2 to 1)	0 (-2 to 1)	-1 (-3 to 1)	0 (-2 to 2)	1 (-1 to 3)
	ES	-0.18 (trivial)	-0.15 (trivial)	-0.36 (small)	-0.20 (small)	0.17 (trivial)
Deceleration zone 4 (m)	Total	17 ± 4	20 ± 4	17 ± 4	17 ± 3	18 ± 5
	First half	9 ± 3	10 ± 3	8 ± 3	9 ± 3	9 ± 3
	Second half	8 ± 2	10 ± 3	8 ± 3	8 ± 3	9 ± 3
	95% CI (first to second)	-1 (-2 to 1)	0 (-2 to 1)	0 (-2 to 1)	-1 (-3 to 1)	0 (-2 to 2)
	ES	-0.35 (small)	-0.13 (trivial)	-0.06 (trivial)	-0.04 (trivial)	0.05 (trivial)

ES, effect size; FB, full-back; FF, full-forward; HB, half-back; HF, half-forward; M, midfielder.

a Data are presented as mean ± SD, mean differences as first half minus second half (95% CI).

b Significance difference from FF.

c Significance difference from M.

Significant decreases were observed in several acceleration and deceleration variables between the first and second halves of play. Significant decrements were observed in the total number of accelerations (*P <* 0.01; mean difference [MD] -9 n; 95% CI -12 to -6; ES, 0.49), the number of accelerations in zones: zone 1 (*P <* 0.01; MD -1 n; 95% CI -1 to -0.02; ES, 0.16), zone 2 (*P <* 0.01; MD -2 n; 95% CI -3 to -1; ES, 0.31), zone 3 (*P <* 0.01; MD -4 n; 95% CI -5 to -2; ES, 0.39), zone 4 (*P <* 0.01; MD -3 n; 95% CI -5 to -2; ES, 0.43). There were significant decrements in the total distance of accelerations (*P <* 0.01; MD -5 m; 95% CI -8 to -3; ES, 0.23), acceleration distance in zone 1 (*P <* 0.02; MD -2 m; 95% CI -4 to -1; ES, 0.18), and acceleration distance in zone 4 (*P <* 0.01; MD -2 m; 95% CI -2 to -0.05; ES, 0.23). There were also significant decrements observed in the total number of decelerations (*P <* 0.01; MD -5 n; 95% CI -7 to -3; ES, 0.43), and the number of decelerations in: zone 2 (*P <* 0.05; MD -1 n; 95% CI -2 to -0.03; ES, 0.25), zone 3 (*P <* 0.01; MD -2 n; 95% CI -3 to -0.05; ES, 0.45), zone 4 (*P <* 0.01; MD -3 n; 95% CI -3 to -1.5; ES, 0.38), and in total distance of decelerations (*P <* 0.01; MD -1 m; 95% CI -2 to -1; ES, 0.16).

**Figure 1.**
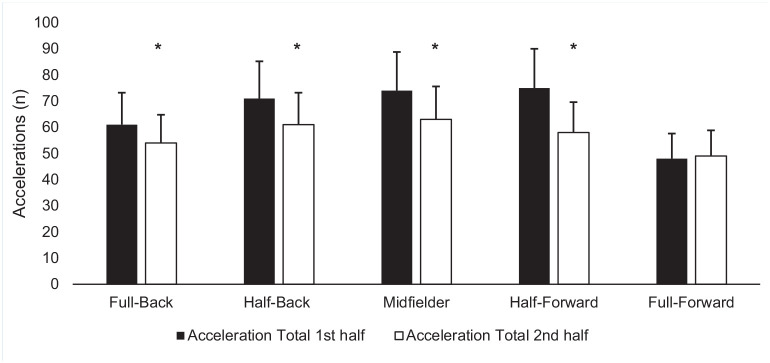
Mean (± SD) acceleration number per half based on position. *Significant difference (*P* < 0.05) between halves.

**Figure 2.**
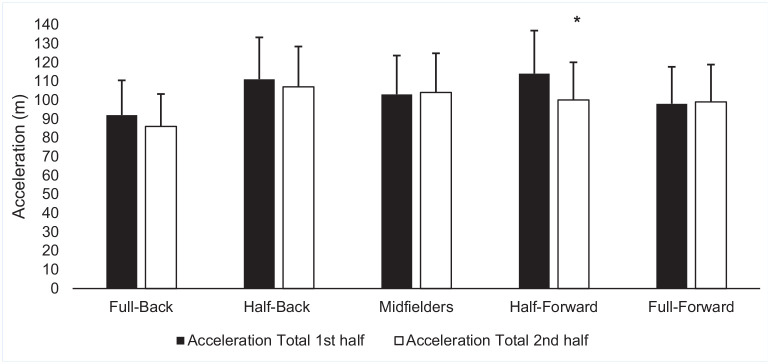
Mean (± SD) acceleration in meters per half based on position. *Significant difference (*P* < 0.05) between halves.

Positional decrements in both acceleration and deceleration variables were observed between halves. Full-backs (*P* < 0.02; MD -7 n; 95% CI -13 to -1; ES, -0.44), half-backs (*P* < 0.01; MD -10 n; 95% CI -15 to -4; ES, -0.68), midfielders (*P* < 0.04; MD -10 n; 95% CI -17 to -4; ES, -0.56), and half-forwards (*P* < 0.01; MD -17 n; 95% CI -24 to -10; ES, -0.85) all experienced decrements in total accelerations between halves (Figure 1). Half-forwards experienced decrements in total number of accelerations in zone 2 (*P* < 0.01; MD -2 n; 95% CI -3 to 0; ES, -0.58). Half-backs (*P* < 0.03; MD -3 n; 95% CI -5 to 1; ES, -0.58) and half-forwards (*P* < 0.01; MD -6 n; 95% CI -8 to -3; ES, -0.65) experienced decrements in total number of accelerations in zone 3. Midfielders (*P* < 0.02; MD -9 n; 95% CI -9 to -1; ES, -0.58) and half forwards (*P* < 0.01, MD -7 n; 95% CI -11 to -3; ES, -0.75) experienced decrements in total number of accelerations in zone 4. Midfielders (*P* < 0.01; MD -6 n; 95% CI -9 to -3; ES, -0.72) experienced decrements in total number of accelerations in zone 4. Half-forwards experienced a significant decrement between halves in acceleration total distance (*P* < 0.01; MD -13 m; 95% CI -20 to -7; ES, -0.65) (Figure 2) and acceleration distance in zone 2 (*P* < 0.02; MD -5 m; 95% CI -9 to 2; ES, -0.44), respectively. Half-backs (*P* < 0.05; MD -3 m; 95% CI -5 to 0; ES, -0.44) and midfielders (*P* < 0.05; MD -3 m; 95% CI -6 to 0; ES, -0.33) both experienced significant decrements in acceleration total distance in zone 4 between halves.

**Figure 3.**
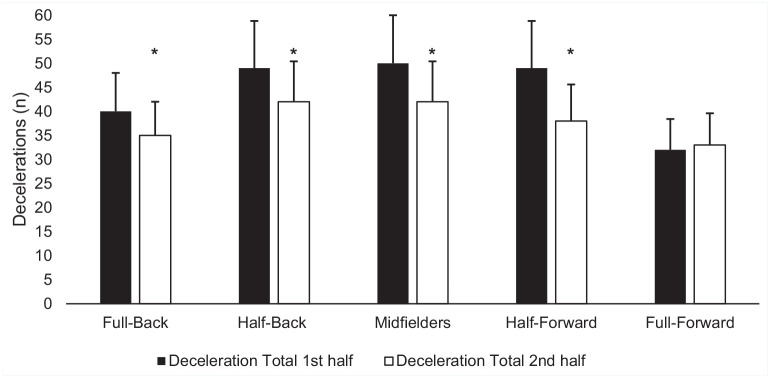
Mean (± SD) deceleration number per half based on position. *Significant difference (*P* < 0.05) between halves.

**Figure 4.**
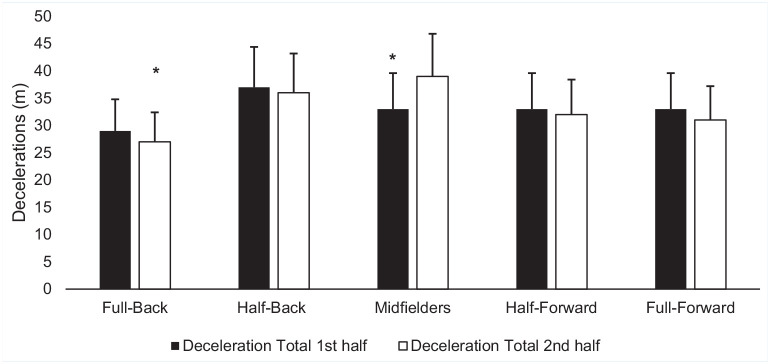
Mean (± SD) decelerations in meters per half based on position. *Significant difference (*P* < 0.05) between halves.

Full-backs (*P* < 0.05; MD -5 n; 95% CI -10 to 0; ES, -0.32), half-backs (*P* < 0.02; MD - 8n; 95% CI -11 to -3; ES, -0.44) and midfielders (*P* < 0.05; MD -8 n; 95% CI -13 to -3; ES, -0.47) all experienced decrements in the total number of decelerations between halves (Figure 3). Half-backs had significant decrements in the number of decelerations in zone 3 (*P* < 0.02; MD -3 n; 95% CI -4 to 0; ES, -0.07). Midfielders (*P* < 0.02; MD -4 n; 95% CI -6 to -2; ES, -0.31), and half-forwards (*P* < 0.01; MD -4 n; 95% CI -6 to -2; ES, -0.46) had significant decrements in the number of decelerations in zone 4 between halves. Full-backs (*P* < 0.02; MD -2 n; 95% CI -3 to 0; ES, -0.38), half backs (*P* < 0.04; MD - 2n; 95% CI -3 to -1; ES, -0.58), midfielders (*P* < 0.05; MD -8 n; 95% CI -3 to -1; ES, -0.37), and half forwards (*P* < 0.01; MD -3 n; 95% CI -4 to -1; ES, -0.88) all experienced decrements in the total number of decelerations in zone 5 between halves. In relation to total deceleration in meters, full-backs (*P* < 0.05; MD -3 m; 95% CI -5 to 0; ES, -0.28) and midfielders (*P* < 0.03; MD -3 m; 95% CI -6 to 0; ES, -0.41) experienced decrements between halves of play (Figure 4). Midfielders experienced a temporal decrement between halves of play in distance in deceleration zone 2 (*P* < 0.02; MD -3 m; 95% CI -5 to 1; ES, -0.36).

The descriptive statistics for variables per position per half are presented in Tables 2 and 3. When comparing positions during match play, the total number of accelerations the half-backs (*P* < 0.05; MD 17 m; 95% CI -1 to 32; ES, -1.75), midfielders (*P* < 0.05; MD 21 m; 95% CI -4 to 38; ES, -1.68), and half-forwards (*P* < 0.05; MD 20 m; 95% CI 3 to 36; ES, -1.38) covered were significantly greater than full-forwards (Figure 1). In acceleration zone 4, the midfielders (*P* < 0.03; MD 9 n; 95% CI 1 to 17; ES, -1.67) and half-forwards (*P* < 0.01; MD n; 95% CI 2 to 18; ES, -1.41) covered a significantly greater number than full-forwards.

In total acceleration distance, half-backs (*P* < 0.05; MD 17 m; 95% CI -1 to 32; ES, -1.75), midfielders (*P* < 0.05; MD 21 m; 95% CI -4 to 38; ES, -1.68), and half-forwards (*P* < 0.05; MD 20 m; 95% CI 3 to 36; ES, -1.38) covered significantly greater distance that full-forwards (Figure 2). In acceleration zone 4, midfielders (*P* < 0.03; MD 9 n; 95% CI 1 to 17; ES, -1.67), and half-forwards (*P* < 0.01; MD n; 95% CI 2 to 18; ES, -1.41) covered a significantly greater number than full-forwards. Midfielders accumulated a significantly greater distance in acceleration zone 4 than full-backs (*P* < 0.05; MD 6 m; 95% CI 1 to 11; ES, -0.57).

In the first half, half-backs (*P* < 0.02; MD 21 m; 95% CI 3 to 39; ES, -0.68), midfielders (*P* < 0.01; MD 26 n; 95% CI 6 to 45; ES, -0.56), and half-forwards (*P* < 0.00; MD 28 n; 95% CI 8 to 47; ES, 0.85) covered a greater acceleration total than the full-forwards. In total number of accelerations in zone 4, midfielders (*P* < 0.05; MD 11 n; 95% CI 1 to 21; ES, -0.72) and half-forwards (*P* < 0.01; MD 13 n; 95% CI 3 to 22; ES, -0.46), performed a significantly greater number than full-forwards. Midfielders also performed a greater number of decelerations in zone 4 than full-forwards (*P* < 0.04; MD 13 n; 95% CI 0 to 14; ES, 0.56). In the second half, midfielders covered significantly greater number of total accelerations than full-forwards (*P* < 0.05; MD 17 n; 95% CI 1 to 34; ES, 0.04).

## Discussion

To the best of the authors’ knowledge, this is the first study to investigate high-intensity movements in intercounty Camogie match play between positions and halves of play. The aim of this study was to compare acceleration and deceleration running profiles of intercounty Camogie players and determine whether there were differences across playing positions and halves of play. The key findings of this research were (1) the number of accelerations and decelerations at different frequencies and intensities varied across the positional lines and (2) there were also significant differences between halves of play. The information provided in this study can better inform practitioners on how to prepare intercounty Camogie players for the acceleration and deceleration demands of competition.

In the current study, intercounty Camogie players performed a total number of 124 ± 29 accelerations and 83 ± 23 decelerations, with the highest number of accelerations in zone 3 (2.21 and 2.9 m s^-2^) 48 ± 13 and the highest number of decelerations in zone 3 (-2.21 and -2.9 m s^-2^), 26 ± 8. These players performed a total distance in accelerations of 202 ± 28 meters and 64 ± 11 in decelerations, with the highest total distance in zone 3 in accelerations of 62 ± 11 meters and 18 ± 4 meters in decelerations in zone 3 and 4 (- >3 m s^-2^). Intercounty LGF players performed a higher number of accelerations 42 ± 6 n (>3 m s^-2^) and decelerations 53 ± 9 n (>3 m s^-2^) in comparison with the Camogie players in this study.^29^ This may be due to the distinct, idiosyncratic nature of each of the female Gaelic games. There were no distances reported in the intercounty LGF study.^29^ In comparison, elite hockey players performed 27 ± 12 accelerations and 40 ± 15 decelerations in match play.^24^ Due to differences in acceleration and deceleration classifications and GPS units utilized, it is difficult to extrapolate these differences between sports.^3^ Furthermore, the differences in variables could be due to the different pitch dimensions, technical and tactical constraints, and game models utilized in the differing invasion-based sports.

As hypothesized, the half-backs, midfield, and half-forward positions accumulated the greatest total accelerations than other positions. Decrements between halves in acceleration and decelerations have been demonstrated previously in LGF,^29^ soccer,^1,28^ and across team-based sports.^20^ The unique positional demands in Camogie may account for these differences. This may explain the full-forward line having the least accumulation of acceleration metrics compared with other positions. Contrastingly, the middle 3 positional lines (half-backs, midfielders, half-forwards) are responsible for transitioning the play between attack and defense, which may be the result of having accumulated more accelerations and decelerations than both the full-backs and full-forwards. In the current study, midfielders covered the greatest number of total accelerations, and accelerations in zone 3 and 4 across all positions. However, the half-back line accumulated the greatest total acceleration distance and distances in zones 3 and 4. Half-back and half-forwards provide attacking support through under and overlapping runs. In addition, these positions may be afforded more space to run towards the goals and into space and transition in and out of possession during match play, which may explain the increased acceleration variables in comparison with other positions.

From a deceleration perspective, the half-backs, midfielders, and half-forwards performed the greatest number of total decelerations and decelerations in zones 3 and 4. The current study differs from the trends reported previously in male hurling.^47^ Camogie midfielders covered more total accelerations and decelerations in zones 3 and 4, whereas the Camogie half-backs covered more total acceleration distance and acceleration distance in zones 3 and 4. This may be due to the distinct contextual demands of intercounty Camogie, including covering space and teammates, recovery running, and initiating attacking play through driving through the middle of the pitch with the slíotar. In LGF, which has similar positional lines to Camogie, the full-back (47 ± 6) and full-forward (47 ± 8) lines accumulated the greatest number of accelerations (>3 m s^-2^); from a deceleration perspective, the full-forwards (59 ± 8) accumulated the greatest number of decelerations (- >3 m s^-2^).^29^ There were no distances reported in the intercounty LGF study. The differences between the sports could be due to the ability to strike the slíotar longer distances with the hurley as opposed to striking the ball with the foot in LGF. Similarly, in elite female soccer, center-backs and full-backs covered less acceleration (>2 m s^-2^) in comparison with the other positional lines, and the wide midfielders and forwards accumulated the greatest number of decelerations (- >2 m s^-2^).^28^

It has been reported that decelerative actions that occur during defensive pressing actions in female invasion-based sports are associated with increased risk of anterior cruciate ligament injuries.^27^ This highlights the need for the middle 8 positions (half-back, midfielder, and half forwards) to receive specific strength and technical training to ensure they can tolerate the acceleration and deceleration demands imposed by intercounty Camogie.

The implementation of acceleration and deceleration training modalities can potentially prepare Camogie players for the unique physiological, biomechanical, and neuromuscular responses of both these high-intensity actions that occur during match play. It has been advocated recently that the utilization of resisted sprint training is an effective modality to elicit specific adaptations to horizontal propulsive forces demonstrated in both early (0-10 m) and late (10-20 m) acceleration phases in invasion-based team sport athletes.^45^ In addition, a combined strength modality approach (maximal and reactive strength) can lead to large improvements in acceleration in female team sport athlete.^23^

The importance of eccentric, isometric, concentric, and reactive strength qualities have been advocated to enhance sports-specific deceleration breaking abilities.^20^ Therefore, to enhance an athlete’s decelerative abilities, practitioners are encouraged to develop both neuromuscular and biomechanical, biomotors qualities. To develop the symbiotic relationship between strength and technical proficiency, practitioners are advised to use both general and specific methods, including eccentric maximal strength, eccentric quasi-isometrics, eccentric landing control, fast eccentric loading, preplanned deceleration movements, reactive deceleration movements, and sports-specific deceleration stimuli.^17^ Recent research has suggested that concentric knee flexor strength was a strong predictor of the total number of decelerations in elite female football players, and that a reduction in knee flexor strength could have a detrimental effect on knee control and stability, increasing injury risk in the later stages of match play.^32^ In addition, it may be prudent of practitioners to consider and monitor the tactically periodized training cycle through manipulation of training drill variables such as pitch dimensions and player numbers during conditioned games.^42^ Furthermore, it is important that each player has the proficiency to effectively accelerate and decelerate at high velocities by improving perception-action coupling in response to dynamic, evolving situations that occur frequently during invasion-based sports.^10^

Due to the intermittent, stochastic nature of invasion-based sports, it has been hypothesized that fatigue in female soccer players could be related to glycogen depletion in the later stages of the second half.^2,25^ Previous research in women’s rugby sevens found that there was a positive correlation between postgame creatine kinase concentrations and distances covered during high-intensity actions such as accelerations and decelerations.^6^ It has been hypothesized that these high-intensity actions can disturb creatine phosphate and adenosine triphosphate turnover, which causes increased accumulation of inorganic phosphate, lactate, and hydrogen ions, which decreases pH level in the muscle and may have an impact on fatigue.^14^ Female team sports athletes who have higher concentrations of skeletal muscle ion transporters have a greater ability to regulate fatigue during the acceleration and deceleration sequences in match play.^32^ Furthermore, specific physical preparation and conditioning may need to be implemented to reduce fatigue decrement across halves in Camogie in relation to these specific acceleration and deceleration movements. It has been suggested that acceleration is a metabolically demanding skill which does not need to occur at high velocities to be physically demanding.^42^ It is hypothesized that decelerative movements induce higher breaking forces and increased rate of neuromuscular fatigue, which could lead to an increased risk of injury.^18,31^ It is therefore the responsibility of the practitioner to design high-intensity activities to ensure the players can skillfully dissipate these forces, develop robust muscular-skeletal systems and take advantage of the repeated bout effect of these high-intensity actions to ensure the players are habituated to the potentially damaging nature of these actions.^31^

A myriad of contextual and tactical factors (in or out of possession), such as game status (win/lose/draw), opponent rank, location, and environmental conditions could also impact the fatigue profile of the players. Previous research in elite male football demonstrated that there was a higher frequency of accelerative and decelerative actions when the team was losing due to the utilization of more defensive breaking actions to order to win back possession.^35^ Coaches could implement in game rotation and substitution strategies in an attempt to delay fatigue mechanisms associated with acceleration and deceleration actions in the second half, especially in the midfield positional group from an acceleration perspective and the half back line from a deceleration perspective.

This study has limitations: first, as the current study was completed with 1 squad of players, the technical and tactical idiosyncrasies of the current team may have influenced the results; therefore, it is not generalizable. The study did not include direct measures of fatigue or technical analysis as part of the data collection process; this should be considered for future research. Second, direction of the game-specific locomotive movement such as evasion, tackling, and collisions were not analyzed. The utilization of GPS units to quantify acceleration and deceleration movement activities has been questioned previously, the data filtering technique utilized and software upgrades used by manufacturer can influence the quality and reliability of the data.^3,38,40^ Arbitrary acceleration and deceleration thresholds were utilized in this study, which could over or underestimate the specific frequency and distance of both measures. Where possible, Horizontal Dilution of Precision (HDOP) and number of satellites should be reported to allow for the evaluation of signal quality.^9^ Unfortunately, the GPS devices utilized in this study did not facilitate the reporting of HDOP and quality of satellite signal. However, data collection was underpinned by previous research recommendations in measuring acceleration including MED, utilization of 10 Hz GPS and the reporting of volume and distance of both acceleration and deceleration metrics across a bandwidth of velocities.^9^

## Conclusion

The number of accelerations and decelerations differed between the positional lines in intercounty Camogie. The half-backs and midfielders covered significantly greater total number of accelerations than full-forwards and midfielders. Half-forwards covered a significantly greater total acceleration distance than full-forwards. In acceleration zone 4, midfielders and half forwards covered a significantly greater number than full-forwards. Midfielders accumulated a significantly greater distance in acceleration zone 4 than full-backs. There were significant decrements observed between halves, in total number of accelerations and accelerations in zones 1 to 4. There were also significant decrements observed in the total distance of accelerations, acceleration distance in zones 1 to 4, total number of decelerations, number of decelerations in zones 2 to 4, and total decelerations distance. To physically prepare Camogie players for the demands of intercounty match-play, it is imperative that they are progressively exposed to acceleration and deceleration stimulus during training both on and off the field of play. Specifically, this research highlights the need for the middle 8 positions (half-back, midfielder, and half-forwards) to receive specific strength and technical training interventions to ensure they can tolerate the acceleration and deceleration demands imposed by intercounty Camogie. The information provided can be utilized by the multidisciplinary sports medicine team to improve performance, guide return to play protocols, and mitigate against the potential injury risks amongst this cohort.
